# The Relative Importance of Globalization and Public Expenditure on Life Expectancy in Europe: An Approach Based on MARS Methodology

**DOI:** 10.3390/ijerph17228614

**Published:** 2020-11-19

**Authors:** Pedro Antonio Martín Cervantes, Nuria Rueda López, Salvador Cruz Rambaud

**Affiliations:** Department of Economics and Business, Universidad de Almería, 04120 Almería, Spain; pmc552@ual.es (P.A.M.C.); nrueda@ual.es (N.R.L.)

**Keywords:** globalization, public expenditure, health outcome, life expectancy at birth, MARS, relative importance, developed countries

## Abstract

Background: There has been a widespread debate about the overall impact of globalization on population, not just economically, but also in terms of health status. Moreover, the current health crisis is going to force governments to review the structure of the public budget to most effectively alleviate the negative economic and health effects on the population. Objective: The aim of this paper is to analyze the relative importance of globalization and the public budget composition—specifically the participation of public expenditure on healthcare, social services and environment in gross domestic product (GDP)—on life expectancy at birth in European countries during the period 1995–2017. Methods: The Multivariate Adaptive Regression Splines (MARS) methodology was applied to analyze the socioeconomic determinants of life expectancy at birth. Results: Our findings show that globalization has no relative importance as an explanatory variable of life expectancy in European countries, while government expenditure on social protection is the most relevant followed by public expenditure on health, gross national income per capita, education level of the population and public expenditure on environmental protection. Conclusion: European strategies intended to impact on health outcome should spend more attention to the composition of public budget.

## 1. Introduction

In Western countries, life expectancy at birth has experienced a remarkable increment in the last century because of the decrease in the probability of death favored by medical and technological advances, reduction of infant mortality rates, changes in nutritional habits and lifestyle, improvement of living conditions and education and the population’s access to health services. Traditionally, three categories of health status determinants have been repeatedly considered in the existing literature, concerning developed countries and focusing on a macro perspective [1]: health resources, lifestyle-related factors and socioeconomic factors. The dimensions interfering in public health outcomes (from a macroeconomic point of view and in the context of developed countries) are summarized in Table 1.

In relation to the first dimension, **health resources**, it is usual to employ either some expressions of health expenditure (such as total expenditure [2,3,4,5,6,7,8,9], public expenditure [10,11,12,13,14,15,16,17,18] or pharmaceutical expenditure [19,20,21]) or some expressions of health resources in physical terms (e.g., the number of doctors and nurses [4,10,22] or the number of beds [11,23]).

A second dimension includes those determinants related to **health habits and lifestyle**, such as the consumption of some substances (e.g., tobacco and alcohol) or the type of diet, represented by the intake of vegetables, fruits, sugar, butter, calories, fats and proteins [4,7,8,19,20].

Thirdly, among the **socioeconomic factors**, most studies in this field consider the gross domestic product (GDP), per capita income [12,22,24,25,26] or some indicators of income distribution [27,28,29]. Education [13,30,31], unemployment [32,33], inflation [34], gross capital formation [35], pollution [12,35,36], environmental quality indicator [37] and financial development [38] are other socioeconomic factors included in this type of empirical research. Additionally, it is usual to consider other specific variables restricted to government expenditures. In this regard, an increasing line of research has analyzed the relationship between social expenditures and health outcome [39,40,41,42,43,44]. In the field of public policy, environmental factors, including greenhouse emissions [9,45,46], have been widely studied as determinants of health outcome. However, the government expenditure aimed at environmental protection has received minimal attention in this type of research, with the exception of the work in [47].

Globalization is another socioeconomic factor which is attracting more and more interest by scholars. Indeed, globalization is a complex and multifaceted phenomenon [48] which has a permanent influence on world economies, increasingly integrated and open to the exterior [49]. From a macroeconomic perspective, a large part of the specialized literature refers to the relationship between globalization and economic growth [50,51,52,53,54,55,56,57,58,59,60,61], per capita GDP or income [62,63,64,65,66]. Another extensive group of investigations is focused on the effects of globalization on government expenditures [67,68,69,70], government revenues [71,72,73] and political budget cycles [74]. Additionally, previous works have also analyzed the link between globalization and either income inequality [75], labor market institutions [76], financial intermediation [77] or human development [78], among others. Recently, another interesting line of research is based on the study of the effect and conditioning factors of foreign direct investment (as a manifestation of globalization) in less developed nations, such as African countries [79,80]. However, the objective of this work is to analyze the association of globalization (and other macroeconomic indicators, such as public expenditures) with a representative indicator of the health status of population, such as life expectancy, in the ambit of European countries.

In this context, there has been a widespread debate about the overall impact of globalization on health status of population. Focusing on a theoretical framework, different mechanisms explaining the relationship between globalization and health outcome can be identified [81,82,83,84]. First, globalization can have a significant effect on public health by facilitating the access to medicines and availability of health treatments. Second, globalization and trade can encourage the consumption of food, soft-drinks, alcoholic beverages and tobacco products by increasing the flow of imports. Third, trade and globalization can impact the organization of the production system of a country, since imports generate competition with domestic firms. Consequently, this may lead to changes in factors which can indirectly influence the health status of population (for instance, wages, employment and working conditions). Fourth, depending on which products and sectors a country’s exports are specialized in, pollution and greenhouse gas emissions can also be altered and, therefore, generate an effect on health status of population. Furthermore, globalization may favor the spreading of infectious diseases, such as coronavirus (COVID-19). There is a rich body of *empirical literature* investigating the relationship between globalization and health outcomes. Most of this type of research concerns developing countries or a mix of developing and developed countries. These studies have employed the economic freedom [85,86], economic openness [87], economic integration [88] or liberalization of trade in services [89] as proxy indicators of globalization to analyze their association with health outcomes. Hauck et al. [90] tested several indicators of trade openness and terms of trade (i.e., measures of the relation between export and import) and other social determinants and Herzer [91] examined the long-run relationship between trade and health. Another group of investigations has considered the KOF Globalization Index (a more refined measure of globalization provided by the KOF (Swiss Economic Institute), which has become the most often used globalization index) [50]. This is a composite indicator which allows measuring different aspects (economic, social and political) of globalization and combining different variables into one index. In this regard, Tsais [92] examined the relationship between the KOF index of globalization and the Human Development Index (HDI) and Bergh and Nilsson [28] analyzed the relationship between the three aforementioned dimensions of globalization and life expectancy. On the other hand, Tausch [93] studied the association between the economic globalization component of the KOF index and the infant mortality. More recently, Jani et al. [94] empirically examined the impact of globalization (measured by the KOF index) on the health status.

Table 2 summarizes the main variables representing dimensions displayed in Table 1. In bold, we highlight the variables employed in this manuscript.

In a scenario of increasing globalization, policy makers in developed and developing countries are interested in improving population health. In addition, the current health crisis is going to force governments to review the structure of their public budget in order to effectively alleviate the negative economic and health effects on the population. Consequently, the main question addressed by this paper is to investigate whether globalization and/or a specific public expenditure affects public health. To do this, the objective of this paper is to analyze the relative importance of globalization and the public budget composition—specifically the participation of public expenditure on healthcare, social services and environment in GDP—in life expectancy at birth in European countries during the period 1995–2017.

## 2. Materials and Methods

### 2.1. Synopsis of the MARS Methodology

The so-called “Multivariate Adaptive Regression Splines” (MARS) [95,96,97] represents a non-parametric alternative to the classic linear regression models. This methodology allows the analysis of nonlinear relationships and complex interactions [98,99,100,101] between the dependent variables and a series of built spline functions on different intervals of a given independent variable [98]. According to Koc and Bozdogan [102], MARS supposes an extension of the original CART models (“Classification and Regression Trees”) [103], whose non-parametric character makes it much more flexible than the strictly linear models [98,101,104,105,106,107,108], as well as more efficient when detecting “outliers” in any data sample [109]. Alreja et al. [104] argued that the main difference between MARS methodology and the linear regression “classical” models stems from the fact that they can unify continuous and categorical data under the same analytical scheme, and they are much easier to interpret and understand. For their part, Samadi et al. [106] confirmed the effectiveness of this approach compared to classical models, since it allows the solution domain to be divided into multiple ranges (of predictive variables or inputs) while detecting a priori hidden relationships between variables, allowing the explicit creation of models. For all these compelling reasons, it was decided to use MARS methodology in this research, taking into account that, as pointed out by Vanegas and Vásquez [101], it consists of a set of techniques still scarcely used in the field of public health, whose nature makes it an important tool for the evaluation of public health indicators, which is the main motivation for this paper.

In addition, its implementation usually improves the results obtained by other types of methodologies such as the logistic models [110,111], multiple linear regression models [112] and, in general, classical Gaussian models that assume a uniform relationship between response and predictors [113]. Likewise, Sephton [114] established that MARS is especially appropriate in the non-linear modeling of time series, while Zhang and Goh [100] showed that it is more reliable in terms of computational efficiency and interpretability than other approaches in the field of artificial intelligence such as Back Propagation Neural Network (BPNN), even outperforming the reliability of the CART models they come from [113].

MARS can be focused within the new scientific paradigm [115] of the “data driven-modeling” [100,116,117], one of the foundations of machine learning techniques, being defined on a bi-objective algorithm (elaborated from a “two-stage process”) [118] in which two different phases are distinguished [108,109,111,119,120]: forward selection and backward deletion. Formally, following Koc and Bozdogan [102] and Zhang and Goh [100], its working-schema can be defined from *Y*, the output or objective-dependent variable response, and $X=(X_{1},\ldots ,X_{j})$, a matrix of *j* input variables (predictors), assuming that the data are generated under an “unknown and true model”. Considering a continuous response, the model would be defined by:

$$Y=f(X_{1},\ldots ,X_{i},\ldots ,X_{j})+e,$$

where *e* is the fitting term error coefficient, and *f*(*X*) is the basis of the built MARS model, composed by splines piecewise polynomial functions, known as Basis Functions (BFs). For the sake of simplicity, it is considered that each BF follows a linear form equal to max(0,x−t) (piecewise linear function) with a given “knot” or breaking point, established in an arbitrary period of time *t*, where the symbol “+” denotes the positive part of the equation:

$$\max{\left(0,x-t\right)}_{+}=\{\begin{array}{l} x-t,\;\mathrm{if}\;x>t\\ 0,\;\mathrm{otherwise} \end{array}$$

and

$$\max{\left(0,t-x\right)}_{+}=\{\begin{array}{l} x-t,\;\mathrm{if}\;x<t\\ 0,\;\mathrm{otherwise} \end{array}$$

The basic philosophy of this methodology [102] is based on forming pairs of predictors of the variable $X_{j},j\in \{1,\ldots ,p\}$, with given knots at each observed value $X_{ij},i\in \{1,\ldots ,n\}$, where *n* is the sample size, so that the set of all possible pairs of variables associated with their corresponding knots, can be defined according to:

(1) $$F=\{{\left(x_{j}-t\right)}_{+},{\left(t-x_{j}\right)}_{+}\;/\;t\in \{x_{1j},x_{2j},\ldots ,x_{nj}\},j\in \{1,\ldots ,p\}\}.$$

Any MARS model can be considered as a re-adaptation of the classic stepwise regression [113], in which the original predictors of the variable are not used, but the set of functions resulting from *F* or its corresponding products (Equation (1)). Therefore, these kinds of models are approximated depending on the equation:

(2) $$f(X)=\beta_{0}+\sum_{m=1}^{M}\beta_{m}\lambda_{m}(X),$$

where the terms $\lambda_{m}(X)$ represent each BF obtained from *F* or the product of two or more functions of this set and $\beta_{0}$ and $\beta_{m}$ denote the intercept terms of the regression, calculated thorough the ordinary least-squares method.

**Figure 1:**
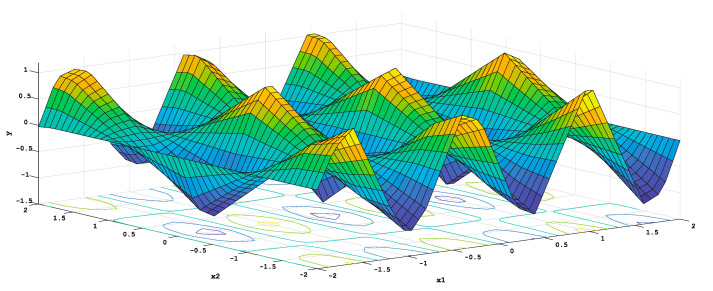
Plot of a piecewise linear MARS model. Source: Own elaboration.

Figure 1 displays a graphic representation of this procedure, using piecewise linear for a two-dimensional function given by the equation $y=f(x_{1},x_{2})=\sin(1.50\pi x_{1})\times \cos(0.50\pi x_{2})$.

In the first stage (or forward phase), the model is purposely constructed with a huge number of BFs which over fit the dataset [118]. Thus, some BFs can erratically contribute to the representativeness of the model, by including non-significant terms which have to be eliminated by means of an iterative process based on the training of the original dataset by using, exclusively, the intercept term $\beta_{0}$ (Equation (2)).

Consequently, only those pairs of BFs which generate the maximum reduction of error will be added to the training process. That is, when considering an initial model composed by *M* basis functions, the next pair of BFs to be included in the model will have the following form [100]:

$${\hat{\beta }}_{M+1}\lambda_{l}(X)\max(0;X_{j}-t)+{\hat{\beta }}_{M+2}\lambda_{l}(X)\max(0;t-X_{j}).$$

Suring the second stage (or backward phase), a pruning operation is performed in order to increase the model accuracy by removing the remaining non-significant terms coming from the forward stage, by reducing the complexity of the model [100] without losing its significance level or its ability to fit the original dataset [118]. In this sense, to specify which BFs have to be included in the final model, MARS implements the *GCV* criterion [97,121]:

(3) $$GCV(\lambda )=\frac{MSRE}{{\left(1-\frac{M(\lambda )}{N}\right)}^{2}}=\frac{\sum_{i=1}^{N}{\left(y_{i}-{\hat{f}}_{\lambda }(x_{i})\right)}^{2}}{{\left(1-\frac{M(\lambda )}{N}\right)}^{2}},$$

where *MSRE* denotes the mean squared residual error and M(λ) is the complexity level of the MARS model given by the number of BFs included in the model (adding its corresponding intercept term) as well as the parameter *d*, known as the “penalty of the model”. Since this parameter can also be considered as a smoothing parameter [122] and in this research the value *d* = 2 was employed to configure a pairwise interactive analysis model [95,99,104,107], the complexity is equal to:

(4) M(λ)=(λ+1)+d×λ.

In any case, the complexity level of the model will not depend specifically on the number of BFs but also on the number of given knots [109]. Alternatively, the *GCV* estimator can be derived from the following equivalence relationship [123]:

$$GCV(\lambda )=\frac{RSS/N}{{\left[1-\frac{M(\lambda )}{N}\right]}^{2}}$$

These criteria must be implemented to select which optimal BFs have to be included in the model, so that a minimum value of this estimator describes a perfect balance between fit and complexity, producing the most appropriate generalization of the built model in terms of accuracy [110]. On the other hand, one of the main features of the *GCV* criterion is its ability to count the number of subsets in which the model is subdivided [124] and so the appearance of each dependent variable is included. In this way, the relative importance of each dependent variable with respect to the independent variable is determined by the number of occasions when they appear in each respective subset.

### 2.2. Data

The aim of this paper is to determine the relative importance of a set of socioeconomic factors in explaining life expectancy at birth in European countries for the period 1995–2017, paying special attention to public expenditures and globalization.

Life expectancy at birth is often used as proxy for health status [4,6,17,19,21,23,28,31,37] due to data limitations and availability over extended periods of time [25] and ease of comparison across countries [10].

Following previous specialized literature, we first considered two “classic” socioeconomic variables included in the empirical research referred to the determinants of health status: *per capita* income [12,22,24,25,26,124] and education [13,30,31,90,125]. Higher incomes are expected to result in better health outcomes by improving access to food, shelter and hygiene [25,34,124]. In the same way, higher levels of education are expected to improve health outcomes by supporting better decision-making abilities and increasing knowledge of preventive care behaviors [30,31,125].

We further used government expenditures, as public expenditure on health. In this point, there is no consensus when identifying the contribution of this type of public resources to health outcomes. The existing literature on this topic identifies positive [10,15,18], moderately negative [14,16] or no significant association [13]. Social protection expenditure by government has also been employed, but, unlike the above public function, most of the thematic literature detects a positive influence of public social expenditure on health status of population [39,40,41,42,43]. The growing importance of environmental issues and the role of the public sector in this area justify the inclusion of public expenditure on environmental protection in our analysis, thus continuing to contribute to the scarce literature in this field [47].

Globalization is measured by the most recent version of the KOF Globalization Index (KOF, Swiss Economic Institute) [126]. This index has been used as a standardized measure of globalization because of its comprehensiveness, as it is an index that, since 1970, has measured the globalization of almost all countries in the world based on three dimensions, namely economic, social and political, by distinguishing two types of measures: *de facto*, focused on the internationalization of economic flows and adjacent activities, and *de iure*, represented by the policies and conditions which facilitate the extension of this process. Therefore, this composite indicator has the advantage of combining different variables and measuring different aspects of globalization in one index. Other indicators of globalization, such as openness to trade and capital flows, offer more limited information. An additional benefit is that the last revision of the KOF index includes new components such as cultural globalization and disentangles trade and financial globalization within the economic dimension of globalization [126]. Another important characteristic of this index is the fact that it offers an individualized image of the impact of globalization, omitting any reference to transactions, trade flows or economic linkages which occur internally within the borders of each country. Previous empirical research suggests a positive association between globalization and a healthier population in developing countries [28,91,92,93,94] whilst other works conclude that there is no relationship between both variables [88,89].

Other socioeconomic factors which have been incorporated into this type of analysis are air pollution, generally represented by emissions of polluting substances [12,25,26], and lifestyle factors, such as smoking; drinking; the intake of vegetables, fruits, sugar, butter, calories, fats and proteins; or even the level of obesity, among others [4,7,8,19,20,127]. Unfortunately, in this work it was not possible to include both types of variables because they are not available for European countries for the entire period 1995–2017.

The definitions, abbreviations and units of the variables employed in this analysis are summarized in Table 3. All data were obtained from the Eurostat database, with the exception of the globalization index, which is compiled by KOF.

More specifically, this dataset is composed of the data from the following 14 European countries over the period 1995–2017: Belgium, Denmark, Germany, Ireland, Greece, Spain, France, Italy, Luxembourg, Netherlands, Portugal, Finland, Sweden and United Kingdom. In total, the seven variables included in the dataset during the aforementioned time horizon determine a pool consisting of 2254 items (14 countries × 23 years × 7 variables).

Table 4 collects the main descriptive statistics of the original dataset from which it is necessary to point out how most of the analyzed variables present a behavior which could be considered as relatively stable throughout the analyzed period, with low values of the standard deviation and the range of variation, with the sole exception of GNI, KOF and LEDU.

Subsequently, in accordance with Montero Granados [128], we rescaled the original data using natural logarithms in order to avoid possible problems related to heteroscedasticity and endogeneity and, likewise, due to the nature of the data, whose level of dispersion advises the use of such transformation. Next, the possible presence of endogeneity in the analyzed time series was analyzed, considering as instruments those variables in which, allegedly, endogeneity could exist: ENVIRO, SOPRO and HEALTH. As can be appreciated in Table 5, the application of the Hausman’s endogeneity test [129] verifies the non-presence of endogeneity in each one of the indicated variables.

Finally, to analyze the reverse causality between the dependent variable and the set of independent variables used in this analysis, the Granger causality test for panel data (“Staked test” or common coefficients) was carried out, by using a number of lags equal to 2. Note that according to the data used, the Dumitrescu–Hurlin version (individual coefficients) would have been more appropriate; however, the number of data used makes this option unfeasible considering the number of constraints imposed by this model. The causal analysis is summarized in Table 6. In fact, the presence of bi-directional causality is not detected, while the Granger causality runs one-way from LEAB to GNI and from SOPRO to LEAB.

### 2.3. Results

The proposed MARS model was implemented in the predefined dataset, being necessary to group the obtained results based on two fundamental aspects, the significance of the employed model and the relative importance of the variables, as well as the overall goodness of the fit. In Table 7, a summary of the main characteristics of the model is displayed, finally composed of 12 BFs which, after the backward deletion phase, were selected from a total of 13 possible alternatives (the intercept term being included in this number). Regarding the importance of the predictors of this model with respect to the dependent variable (LEAB), five of six initially considered were selected (see Table 1), estimating a null or erratic representativeness of the variable KOF, being the importance of the rest of independent variables (from the highest to the lowest) the following: SOPRO (1); HEALTH (2); GNI (3); LEDU (4); and ENVIRO (5).

Thus, the optimal MARS model with its corresponding BFs can be presented in the subsequent form:

$$\begin{array}{c} \beta_{0}+\beta_{1}\max(\mathrm{SOPRO}-3.09104)+\beta_{2}\max(\mathrm{SOPRO}-3.17388)+\beta_{3}\mathrm{ENVIRO}\max(\mathrm{SOPRO}-3.09104)\\ +\beta_{4}\max(\mathrm{LEDU}-4.24276)\max(\mathrm{SOPRO}-3.09104)+\beta_{5}\max(\mathrm{LEDU}-4.27388)\max(\mathrm{SOPRO}-3.09104)\\ +\beta_{6}\max(\mathrm{SOPRO}-3.09104)\max(\mathrm{HEALTH}-1.96009)+\beta_{7}\max(\mathrm{SOPRO}-3.09104)\max(\mathrm{HEALTH}-1.88707)\\ +\beta_{8}\max(\mathrm{SOPRO}-3.09104)\max(\mathrm{GNI}-3.36881)+\beta_{9}\max(\mathrm{SOPRO}-3.09104)\max(3.36881-\mathrm{GNI})\\ +\beta_{10}\max(\mathrm{LEDU}-4.24276)\max(\mathrm{SOPRO}-3.09104)\max(\mathrm{GNI}-3.43234)\\ +\beta_{11}\max(\mathrm{SOPRO}-3.09104)\max(\mathrm{HEALTH}-1.93152)\max(\mathrm{GNI}-3.36881) \end{array}$$

Table 8 reflects the metrics GCV and RSS, based on the number of times in which each independent variable appears in each subset on which the model has been defined.

**Figure 2:**
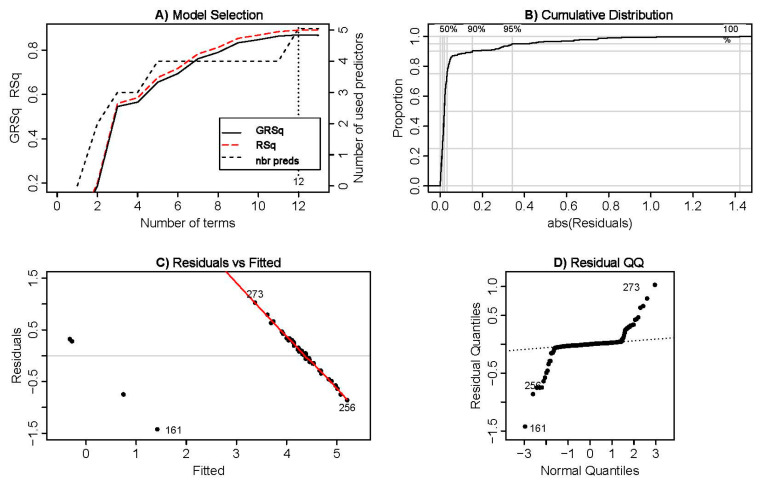
Model Selection. Source: Own elaboration. GRSq, Generalized coefficient of determination; RSq, Coefficient of determination; nbr preds, Number of used predictors.

Figure 2 displays the most important characteristics of the selected model according to different adjustment measures. In this sense, Figure 2A collects the evolution of the RSQ and GRSq measures, evaluating the model performance: both almost converge with 13 optimal predictors (or BFs included in the model). Figure 2B exhibits the cumulative function resulting from the implemented model based on the value of each residual term in absolute values: the goodness of fit manifests itself again that, starting from a relatively low value of the model residuals, the explanatory capacity of the curve distribution can be considered high (around 90%). Finally, analyzing the residuals associated with the model, Figure 2C,D shows that the constructed model presents a quite acceptable fit (in accordance to the RSq and GRSq values), detecting the presence of sample “outliers” (data points: 161, 256 and 273), which were not included in the final adjustment of the MARS model after the backward deletion process.

## 3. Discussion

This study determined the relative importance of globalization and the composition of public budget in 14 European countries during the period 1995–2017 by using the MARS methodology. The results conclude that public expenditures on social protection and healthcare are the variables with the greatest relative importance in explaining life expectancy at birth, followed by per capita gross national income, the educational level of the population and public expenditure on environmental protection. On the contrary, globalization has no relative importance in European countries.

With respect to the composition of public budget, social protection expenditure is the most relevant determinant of health outcome in this study. Our results confirm most of the existing literature concluding that greater social protection expenditure is associated with a better health status [39,40,41,42,43]. On the other hand, higher social expenditures (e.g., family, unemployment, incapacity, old age and active labor market programs, among others) may improve health status by reducing poverty, promoting access to early childhood programs and providing social benefit coverage which may reduce chronic stress related to, for example, cardiovascular disease. Additionally, the results show that public expenditure on healthcare is in the second place in terms of relative importance in explaining life expectancy. In this sense, there is no consensus in specialized literature when identifying the contribution of public resources to health outcomes. Some authors have identified a positive significant contribution on health outcomes related to public healthcare expenditure [10,15,18], although in some cases no significant impact has been detected [13] and, in other works, this effect is moderately adverse [14,15,16]. In the beginning, higher public health expenditure could be associated with significant gains in health status of population, even though this association is not applicable in high-expenditure countries [130]. Moreover, the authors of [131] concluded that there is a point of saturation at which increases in public health expenditure do not necessarily imply increases in life expectancy. This may merely reflect an inflated administration, expensive technologies, poor comparative effectiveness or personal financial advantages for interest groups or individuals [42].

Our findings related to per capita income, the third most relevant socioeconomic factor in this research, are consistent with previous literature [25,34,124]. In effect, higher per capita income affects health status improving nutrition, access to health care and working conditions. In this way, these results confirm the Preston’s [132] curve which relates national income with average life expectancy at birth for a range of countries at one point in time and shows that people living in rich countries on average live longer than people in poor countries.

The level of education also has a relative importance in explaining life expectancy, confirming previous research [30,31,125]. Education is generally considered a social determinant of health because of three main reasons [90]: more education improves health; better health leads to more education; and this association between education and health can be indirectly explained by additional variables relating to the household and wider environment, such as parental education.

The available evidence on the impact of public environmental protection expenditures on life expectancy from a macro approach is more scarce [47]. For this reason, this work has included this factor in this type of research by using macro data. Our findings show that environmental expenditures are one of the least important socioeconomic factors related to life expectancy at birth [47]; this does not mean that this type of public expenditure has no effect on health status, but this item has less relevance than other factors such social and healthcare expenditures.

Our results conclude that globalization has no relative importance in explaining life expectancy at birth in European countries. Similarly, Bussmann [88] and Umaña-Peña et al. [89] found empirical evidence about the non-relevance of globalization on health status. More specifically, Tausch [93] suggested that globalization only improves healthcare status for underdeveloped countries, which could explain our results referred to European (not underdeveloped) countries and the relative unimportance of globalization in terms of health improvement. In the same vein, the authors of [91,94,133] pointed out that the positive association between globalization and a healthier population would be limited to developing countries. In this regard, this positive relationship between globalization and health in the least developed countries can be explained by increasing the easy supply of life-saving drugs, improving the institutional features of an economy which may influence the lifestyles of masses and enhancing the use of friendly-environment methods of production by firms [133]. From a different point of view, Mourão [74] also showed the higher (lower) exposure of developing (developed) countries to globalization. Specifically, they concluded that “globalization tends to increase government size in new democracies and developing countries and contribute to sharper political economic cycles”. However, “mature democracies and developed countries can be achieved without significant reactive characteristics in their political cycles to higher levels of trade openness”.

Summarizing, the relative importance of globalization can be reduced or even annihilated when considering developed countries, as is the case of the sample of countries analyzed in this work. This can be explained by the fact that in underdeveloped countries most of the poor population does not have access to healthcare. In this context, globalization (mainly economic globalization) allows citizens to have access to primary healthcare, which can have a greater impact on health status than in more developed countries with more effective health care systems and better health population indicators. Furthermore, it is worth noting that, in the most developed countries, life expectancy has less room for improvement due to its “natural” cap, as it is usually much higher than in the underdeveloped world.

The results should be interpreted taking the limitations of our study in mind. First, in the analysis of the determinants of life expectancy, not only does per capita income matter, but also the existence of inequalities in its distribution. With respect to inequality issues, Leitner [134] argued that income inequality in the European Union countries influenced population health outcomes, such as life expectancy, infant mortality rate and standardized death rates. Thus, it could be worthwhile to consider these topics in future research. Another restriction of this study is that lifestyle factors (e.g., smoking, obesity or drinking) and other indicators of quality of life (such as stress and working conditions) have not been considered because they are not available for the sample of European countries analyzed in this study and for a consistent period. Future research should include this type of information.

Specifically, the main contributions of this manuscript are the following ones: First, this paper attempts to fill the gap of the empirical research on the relationship between globalization and health status referred to European countries which is less extensive than that focused on developing countries. Second, to the extent of our knowledge, this is the first time that MARS methodology has been applied to analyze the socioeconomic determinants of life expectancy at birth. This methodology allows the analysis of nonlinear relationships and complex interactions between the dependent variable and a series of built spline functions on different intervals of a given independent variable.

**Figure 3:**
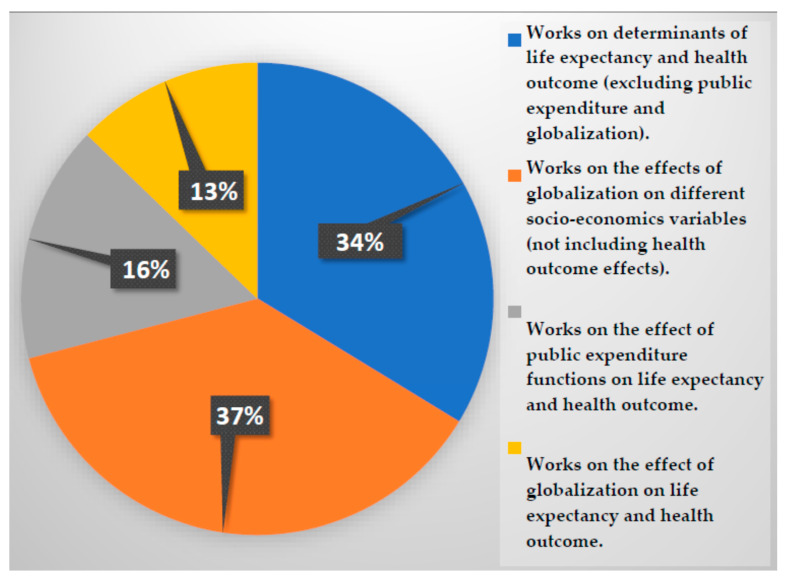
Pie chart of the main groups of contributions related to the literature reviewed in this study. Source: Own elaboration.

Summarizing, this paper aims to enrich the specialized literature by introducing not only globalization as an explanatory variable of life expectancy, but also the composition of public health budget. To contextualize our manuscript in the existing literature, we carried out a cumulative analysis which includes the main lines of research from 1969 to 2020. To this end, four main blocks are distinguished: (i) works on determinants of life expectancy and health outcome (excluding public expenditure and globalization); (ii) works on the effect of globalization on different socioeconomic variables (not including health outcome effects); (iii) works on the effect of public expenditure functions on life expectancy and health outcome; and (iv) works on the effect of globalization on life expectancy and health outcome (see Figure 3).

Due to the importance of government expenditure and the sustainability of public finances from a macroeconomic perspective, new public expenditure items should be incorporated into this type of analysis with the aim of advising policy makers in the allocation of budgetary resources. The environmental protection function is the great unknown among budget expenditures with respect to its relationship to life expectancy, since it has rarely been analyzed in this field. Further research is therefore needed to relate public environmental programs and health status.

The extent to which socioeconomic factors can affect health status of citizens is a topic which has received more attention nowadays as the result of the global pandemic caused by COVID-19. Consequently, it is also necessary to include in this type of research the factors related to the risk and intensity of the spread of infectious diseases and pandemics.

## 4. Conclusions

Employing the MARS methodology, this study classified the socioeconomic factors according to their relative importance when explaining life expectancy at birth in European countries. Our findings show the non-relevance of globalization on public health in this area of developed countries. Government expenditures on social protection and healthcare are the variables with the greatest relative importance in explaining life expectancy at birth, followed by per capita income, the educational level of the population and the public expenditure on environmental protection.

With respect to the causal analysis, the Granger causality test did not detect the presence of bi-directional causality, while it showed one-way causality from life expectancy to gross per capita national income and from expenditure on social protection to life expectancy.

The main political recommendation is that European strategies intended to impact on health outcome should focus not only on improvements in “traditional” variables such as per capita income. More attention must be spent on the composition of the public budget, since, unlike what might be expected, public social expenditures seem to be more relevant for health outcomes than healthcare expenditures.

## Figures and Tables

**Table 1:** Dimensions of the determinants of health outcomes. Source: Own elaboration.

Dimensions of Determinants (Context: Developed Countries)	
Explaining	Explained
Healthcare resources	Health outcomes
Lifestyle-related factors	
Socioeconomic factors	

**Table 2:** Determinants of health outcomes by dimension. Source: Own elaboration.

Variables	
Explaining (by Dimension)	Explained
Healthcare resources:	**Life expectancy at birth (LEAB)** (mortality rate or infant mortality)
**Health expenditure** (total or **public**) **(HEALTH)** Pharmaceutical expenditure Number of doctors and nurses Number of beds	
Health habits and lifestyle:	
Consumption of substances (tobacco and alcohol; among others) Type of diet	
Socioeconomic variables:	
Gross domestic product **Per capita income (GNI)** Income distribution **Education (LEDU)** Unemployment Inflation Gross capital formation Pollution Environmental quality and public **environmental protection expenditure (ENVIRO)** Financial development **Globalization index (KOF)** **Social expenditure** (total or **public**) **(SOPRO)**	

**Table 3:** Description of the variables considered for the MARS model. Source: Own elaboration from data obtained from Eurostat and the last revision of the KOF Index [126].

Variable		Definition	Units
LEAB	Dependent variable	Life expectancy at birth	Years
GNI	Independent variable	Gross per capita national income at current prices	Thousands of Euros
LEDU	Independent variable	Percentage of population with upper secondary, post-secondary non-tertiary and tertiary education	Percentage of total population
KOF	Independent variable	KOF Globalization Index	Weighted index average
HEALTH	Independent variable	General government expenditure on health	Percentage of GDP
SOPRO	Independent variable	General government expenditure on social protection	Percentage of GDP
ENVIRO	Independent variable	General government expenditure on environmental protection	Percentage of GDP

**Table 4:** Descriptive Statistics of the dependent and independent variables. Source: Own elaboration.

Variable	Mean	St. dev.	Variance	Coef. var.	Minimum	Maximum	Range
LEAB	79.74	1.845	3.404	2.31	75.3	83.5	8.2
LEDU	62.493	14.102	198.857	22.57	19.3	82.3	63
ENVIRO	0.7792	0.3439	0.1183	44.14	0.2	1.7	1.5
SOPRO	18.109	3.685	13.579	20.35	9.5	25.5	16
HEALTH	64.149	10.745	11.546	16.75	3.7	8.9	5.2
KOF	84.089	4.458	19.87	5.3	68.112	91.313	23.201
GNI	29.872	10.954	119.988	36.67	9.151	65.663	56.511

**Table 5:** Hausman test. Source: Own elaboration.

Dependent variable: LEAB				
Instrumented: ENVIRO SOPRO HEALTH				
Instruments: const LEDU KOF GNI				
	Coefficient	St. Dev.	*t*-statistic	*p*-value
Constant	69.2710	1.91263	36.22	<0.0001
ENVIRO	6.60997	2.13933	3.090	0.0022
SOPRO	0.465163	0.139315	3.339	0.0009
HEALTH	0.483982	0.500587	0.9668	0.3344
Mean dep. variable	79.74049		St. dev. dep. variable	1.845102
Sum squared residuals	2310.229		S. E. of regression	2.695342
R-squared	0.061202		Adjusted R-squared	0.052345
F(3,318)	10.20653		*p*-value (of F)	1.96 × 10^−6^
Bayesian inf. crit.	4068.865		Akaike inf. crit.	8145.730
Schwarz inf. crit.	8160.828		Hannan-Quinn Inf. Crit	8151.758
Hausman Test				
Null hypothesis [OLS estimates are consistent]:				
Asymptotic test statistic: Chi-square (3) = 34.787, with a *p*-value = 1.35123 × 10^−7^				

**Table 6:** Causal relationship: explained vs. explicative variables. Source: Own elaboration.

Null Hypothesis:	Obs.	F-Statistic	Prob.
LEAB does not Granger cause ENVIRO	280	1.70333	0.1840
ENVIRO does not Granger cause LEAB		1.90749	0.1504
LEAB does not Granger Cause GNI *	280	8.71157	0.0002
GNI does not Granger Cause LEAB		0.35845	0.6991
LEAB does not Granger Cause HEALTH	280	0.62930	0.5337
HEALTH does not Granger Cause LEAB		2.58102	0.0775
LEAB does not Granger Cause KOF	280	2.26417	0.1059
KOF does not Granger Cause LEAB		0.11235	0.8938
LEDU does not Granger Cause LEAB	280	1.58716	0.2064
LEAB does not Granger Cause LEDU		0.03430	0.9663
SOPRO does not Granger Cause LEAB *	280	4.55385	0.0113
LEAB does not Granger Cause SOPRO		1.84299	0.1603

* the null hypothesis is rejected.

**Table 7:** Summary of the predictive model obtained through the implemented MARS approach. Source: Own elaboration.

Corresponding Equations of the Model		
	Equation	Coefficients
Intercept	-	4.37906
**BFs**		
BF1	max(SOPRO-3.09104)	22.63448
BF2	max(SOPRO-3.17388)	−30.62018
BF3	ENVIRO max(SOPRO-3.09104)	−8.80387
BF4	max(LEDU-4.24276) max(SOPRO-3.09104)	−967.90106
BF5	max(LEDU-4.27388) max(SOPRO-3.09104)	1052.04227
BF6	max(SOPRO-3.09104) max(HEALTH-1.96009)	−805.12841
BF7	max(SOPRO-3.09104) max(HEALTH-1.88707)	366.06836
BF8	max(SOPRO-3.09104) max(GNI-3.36881)	−199.00359
BF9	max(SOPRO-3.09104) max(3.36881-GNI)	−62.16061
BF10	max(LEDU-4.24276) max(SOPRO-3.09104) max(GNI-3.43234)	638.65821
BF11	max(SOPRO-3.09104) max(HEALTH-1.93152) max(GNI-3.36881)	1287.04012
**Main Features of the Model**		
GCV:	0.03863691	
RSS:	10.33624	
GRSq:	0.8691754	
RSq:	0.8906307	
Terms (BFs):	Selected 12 of 13 (including the intercept term)	
Predictors:	Selected 5 of 6	
Predictors importance (in order):	SOPRO, HEALTH, GNI, LEDU, and ENVIRO (KOF represents an “unused” predictor)	

GCV, Generalized Cross Validation; RSS, Residual Sum of Squares; GRSq, Generalized coefficient of determination; RSq, Coefficient of determination.

**Table 8:** Relative importance of independent variables vs. dependent variable. Source: Own elaboration.

Variable	Number of Subsets	GCV	RSS
SOPRO	11	100.0	100.0
HEALTH	10	98.9	98.5
GNI	9	87.3	86.4
LEDU	9	61.0	60.9
ENVIRO	2	17.1	19.1
KOF	-	-	-
